# Innovative Alcoholic Drinks Obtained by Co-Fermenting Grape Must and Fruit Juice

**DOI:** 10.3390/metabo9050086

**Published:** 2019-04-30

**Authors:** Daniela Fracassetti, Paolo Bottelli, Onofrio Corona, Roberto Foschino, Ileana Vigentini

**Affiliations:** 1 Department of Food, Environmental and Nutritional Sciences, Università degli Studi di Milano, Via G. Celoria 2, 20133 Milan, Italy; daniela.fracassetti@unimi.it (D.F.); paolo.bottelli@gmail.com (P.B.); roberto.foschino@unimi.it (R.F.); 2 Department of Agricultural, Food and Forest Sciences, University of Palermo, Viale delle Scienze, 90128 Palermo, Italy; onofrio.corona@unipa.it

**Keywords:** fruit wines, food innovation, yeasts, secondary metabolites, grape must, kiwi juice

## Abstract

In this study, Cabernet Sauvignon and Chardonnay musts, and fruit juices from cherry, kiwi, peach, and strawberry were co-fermented with *Saccharomyces cerevisiae* EC1118 and *Torulaspora delbrueckii* UMY196 at two different proportions (80:20 (*v*/*v*) and 60:40 (*v*/*v*)). The most pleasant fruit-based drink was obtained with Cabernet Sauvignon must and kiwi juice in a proportion of 60:40 and fermented with *T. delbrueckii*. This beverage was produced in higher volume to simulate a scale-up, and the aromatic profile, sensory description, and consumer acceptability were determined. The most powerful odorants of the kiwi-based drink were ethyl octanoate, phenylethanal, ethyl hexanoate, vinyl-guaiacol, benzaldehyde, and nonanal, for which the odor activity values were 21.1, 3.3, 2.6, 2.2, 1.9, and 1.6, respectively. These findings were in accordance with the sensory analysis, since the emerged descriptors were fruity (ethyl octanoate), honey and floral (phenylethanal), apple and peach (ethyl hexanoate), and citrus (nonanal). The consumers judged the kiwi-based drink acceptable (67%) and 39% of them would buy it. The reliable fermentation of a grape must/fruit juice was demonstrated. The kiwi-based drink represents an innovative and pleasant beverage with a positive impact on sustainability as its production can limit the loss of fresh fruits, as well as contribute to the enological field.

## 1. Introduction

Innovation in the sector of alcoholic beverages using sustainable approaches is a challenge from both environmental and productive points of view. Indeed, possible solutions consider several aspects including waste re-conversion or re-use, social impact (i.e., reduction of food loss, production of low-alcohol beverages), and economic advantage (i.e., decrease in wine consumption, unsold wine). Wine is an alcoholic beverage obtained by yeast fermentation of a purely grape must of *Vitis vinifera* vine species. The art of winemaking started back ca. 6000–5800 before Christ (BC) during the early Neolithic Period in Georgia in the South Caucasus region [1], while alternative alcoholic beverages from hawthorn fruit, rice, and honey mead were already produced as early as ca. 7000 BC in ancient China [2]. The resulting alcoholic products from fruits other than grapes are called “fruit wines” and they show differences in taste, nutritive values, and health benefits [3].

In the last 15 years, global wine consumption went up and down [4]. Europe is consuming less and less wine, while the wine consumption in young producing areas is still growing. New alternative wines are appearing on the market and they are cheap and easy-drinking. Examples are the “flavored” wines (red lollipop, peach, grapefruit, mandarin, or black currant) with medium alcohol content (from 8% to 10.5%) obtained by blending wines and fruit juices or flavoring wines with artificial or natural aromas. The main consumers of these products are younger people between 18 and 34 years old, with 33% drinking flavored wines as an aperitif.

Fruit wines are produced from fruit juices other than grape, such as apples, apricots, berries, cherries, plums, strawberries, oranges, mangoes, bananas, and pineapples [5,6], as well as autochthonous Brazilian fruits [7]. Moreover, the production implies the use of fruit juice concentrates, which allows increasing ethanol yield and taste, aroma, and functional features [8]. The production of alcoholic beverages obtained from the co-fermentation of grape must and fruit juice is yet to be investigated. Advantages of this approach are ascribable to an ever-growing rate of global food consumption, whereby the food supply production needs to fulfil all requests in a sustainable way in terms of environmental soundness, social equity, and economic feasibility. The formulation of new mixed-fruit alcoholic beverages could represent a reduction in fruit surplus and post-harvest fruit loss, and it could positively contribute to the economy of the existing wine industry. The reduction of food loss and wastes is gaining increasing importance [9] for increasing the food chain sustainability, even with the production of novel foods. The fermentation of fruit juices using selected yeasts can yield final products enriched in novel bio-functional compounds not found in traditional wines [3].

This study aimed to investigate, from microbial, chemical, and sensory points of view, the co-fermentation of grape musts and fruit juices obtained from cherries, kiwi, peaches, and strawberries. Alcoholic fermentations were carried out inoculating either *Saccharomyces cerevisiae* or the non-*Saccharomyces* species, *Torulaspora delbrueckii*, in four blends of grape musts (Chardonnay and Cabernet Sauvignon) and juices in different proportions. Consumer acceptability was also evaluated. The novel products could be produced using the surplus of some agriculture systems, such as spring/summer fruits and grape.

## 2. Results and Discussion

### 2.1. Flask Trials

#### 2.1.1. Fermentation Trends and Chemical Composition

**Figure 1:**
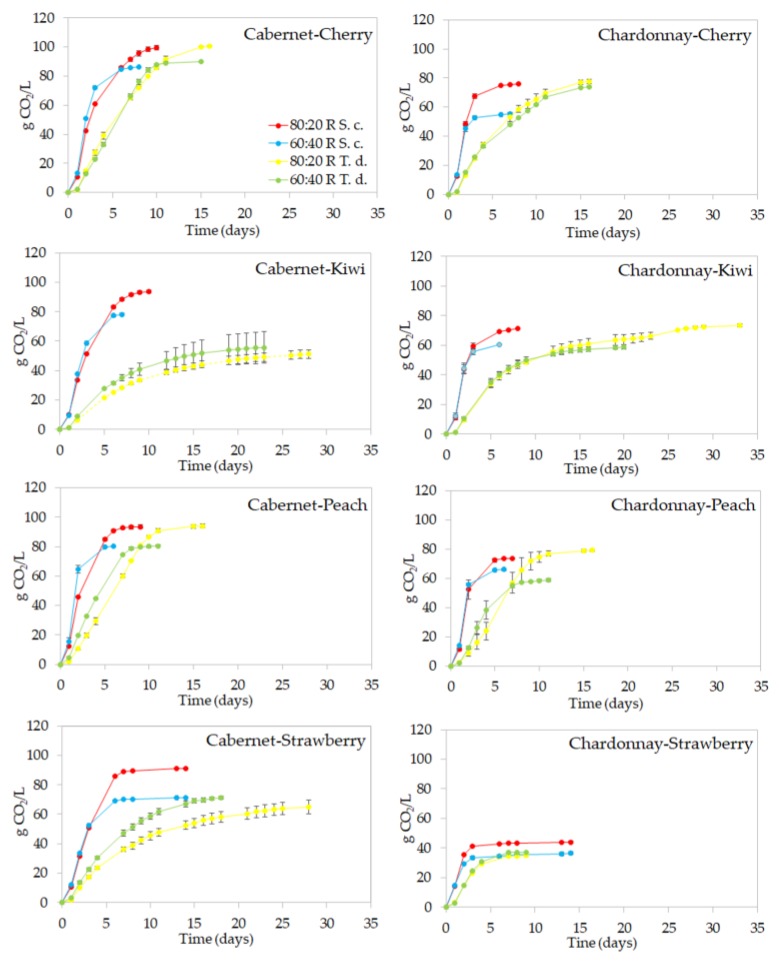
Alcoholic fermentation trends for the trials in flasks of different combinations must/fruit juice inoculated with *Saccharomyces cerevisiae* EC1118 (S. c.) and *Torulaspora delbrueckii* UMY196 (T. d.). Error bars indicate the standard deviation among replicates.

The trend of alcoholic fermentation (AF) was monitored for each must/fruit juice mix. *T. delbrueckii* showed a lower fermentative vigor in comparison to *S. cerevisiae* (Figure 1) as previously found in grape must fermentation [10]. *S. cerevisiae* started the AF in 24 h, producing the following averages: (i) from Cabernet musts/fruit-based mixes, 10.90 ± 0.40 and 12.55 ± 0.70 g CO_2_/L in 80:20 and 60:40 proportions, respectively; (ii) from Chardonnay musts/fruit-based mixes, 12.35 ± 0.95 and 13.55 ± 0.08 g CO_2_/L in 80:20 and 60:40 proportions, respectively. The AF started in 48 h with *T. delbrueckii* reaching similar values of CO_2_/L as the corresponding *S. cerevisiae* trials: (i) from Cabernet musts/fruit-based mixes, 10.49 ± 3.35 and 13.84 ± 4.39 g CO_2_/L in 80:20 and 60:40 proportions, respectively; (ii) from Chardonnay musts/fruit-based mixes, 11.55 ± 2.73 and 13.26 ± 2.16 g CO_2_/L in 80:20 and 60:40 proportions, respectively. While the AF carried out with *S. cerevisiae* ended in 3–10 days, depending on the grape/fruit-based mix, *T. delbrueckii* completed the AF in a longer time (9–33 days) (Figure 1). *S. cerevisiae* showed a comparable fermentative profile for all types of fruit. In the case of *T. delbrueckii*, the fermentative trend with kiwi was significantly slower, regardless of the grape–kiwi juice proportion and combination. Since all inocula were standardized at the same cell concentration (1 × 10^6^ colony-forming units (CFU)/mL) (data not shown), this result needs further investigation.

The residual sugars were about 3 g/L, except for the kiwi-based drinks obtained with Cabernet Sauvignon must and fermented with *T. delbrueckii* (Table 1). A higher content of residual sugars and a slower fermentation can have a positive impact on the aromatic profile [10]. The ethanol yield was comparable between *T. delbrueckii* and *S. cerevisiae* (Table S2, Supplementary Materials); this is of interest for the production of quality wines where the former species could be used as a starter culture [11].

**Figure A1:**
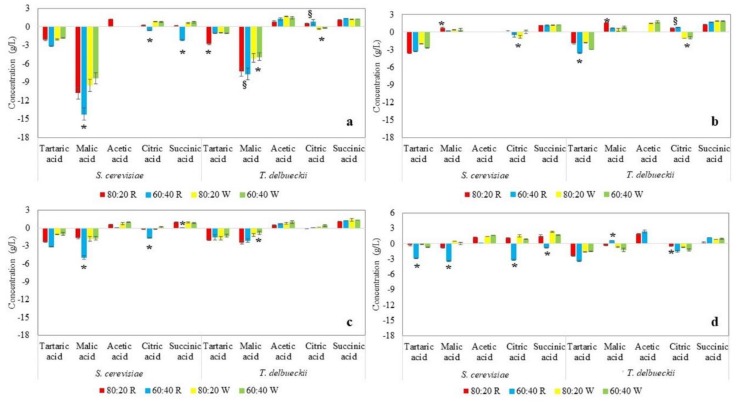
Evolution of organic acids (g/L) at the end of fermentations for the drinks produced in the flask experiment for (**a**) cherry, (**b**) kiwi, (**c**) peach, and (**d**) strawberry juices. Data were calculated as the difference between sampling at the end of alcoholic fermentation and at the initial time. Error bars indicate the standard deviation among the replicates.

At the end of AF, the concentrations of sugar, ethanol, and organic acids, as well as the pH and total acidity, were determined; the characteristics of all final products are summarized in Table A1 and Figure A1 (Appendix A). The decrease in tartaric acid found for the cherry-, peach-, and kiwi-based drinks could be due to either salification or precipitation phenomena [12]. A drop of malic acid was detected particularly for the cherry-based drinks. This finding needs further investigation since the contribution of *S. cerevisiae* in malic acid decrease can be excluded because it lacks a specific system for malic acid transport. An increase in total acidity was found, especially in trials where *T. delbrueckii* was inoculated. This could be due to the higher concentrations of succinic acid responsible for an increase in titratable acidity during fermentation [13]. *T. delbrueckii* was a higher producer of succinic acid than *S. cerevisiae* during the fermentation of must [11]; further studies will be carried out to clarify the behavior of *T. delbrueckii* during the co-fermentation of grape must and fruit juice.

#### 2.1.2. Sensory Analysis

The beverages obtained from co-fermenting Cabernet Sauvignon and cherry juice were particularly unpleasant with both yeasts (scores of overall acceptability <0.5/10) (Figure S1A, Supplementary Materials) due to the note of “chemical–medicinal” flavors. Drinks produced with Chardonnay and cherry juice received a better overall acceptability score, albeit still low (3/10) (Figure S1B, Supplementary Materials).

**Figure 2:**
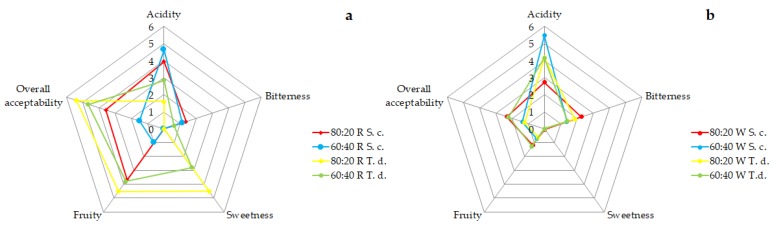
Gustatory profile of kiwi-based drinks for the trial in flasks fermented with (**a**) *Saccharomyces cerevisiae* EC1118 (S. c.), and (**b**) *Torulaspora delbrueckii* UMY196 (T. d.) with red (R) and white (W) grape musts. Data were obtained from medians of the scores indicated by the judges.

Most appreciated grape/fruit-based drinks resulted from with the fermentation of kiwi and Cabernet Sauvignon must, at the two proportions of 80:20 and 60:40, inoculated with a pure culture of *T. delbrueckii*. Indeed, these products received an average score of 5.4/10 and 4.7/10, respectively (Figure 2A). Their pleasantness was associated with a high perception of fruity aromas (4.5/10 and 3.8/10, respectively) and sweetness (4.5/10 and 2.8/10, respectively). In agreement with McMahon and collaborators [14], the presence of sugars allows a decrease in bitterness and acidic taste perception. On the contrary, drinks from Chardonnay must and kiwi juice resulted unpleasant (Figure 2B).

A generally low taste score (2.4/10) was assigned to drinks produced with peach juice (Figure S1C,D, Supplementary Materials). The least pleasant combinations were obtained with Chardonnay must at an 80:20 proportion (0.64/10) fermented with both yeasts, separately, for which the descriptor of “chemical–solvent” flavor was indicated.

For the strawberry-based drinks, the highest scores were related to the perception of acidity. Although the fruity note was also perceived, the overall acceptability was very low (0.45/10) (Figure S1E,F, Supplementary Materials). In combination with Cabernet Sauvignon, it emerged that the bitterness negatively affected the overall acceptability.

Based on the sensory evaluation, the drink from the Cabernet Sauvignon must/kiwi juice mix at the proportion of 60:40 fermented by *T. delbrueckii* was selected as the most promising novel beverage and it was replicated in batches (0.6 L) and in microvinification (4 L).

### 2.2. Batch Experiment

#### 2.2.1. Alcoholic Fermentation Trend

The Cabernet Sauvignon/kiwi blend had the chemical characteristics shown in Table 2. The yeast inoculum resulted similar to the one performed during the preparation of flasks (5.8 ± 1.6 × 10^6^ vs. 6.2 ± 1.6 × 10^6^ CFU/mL). A higher amount of g CO_2_/L was developed in 48 h during the fermentation in batch in comparison to the fermentation in flask (28.5 ± 5.4 vs. 8.97 ± 0.53 g CO_2_/L produced in two days). The faster alcoholic fermentation could be due to the sampling, which was carried out by opening the bottles, leading to a possible aeration of must. This could favor the synthesis of essential fatty acids and sterols required for yeast replication [15] and, consequently, the fermentation rate could increase.

**Figure 3:**
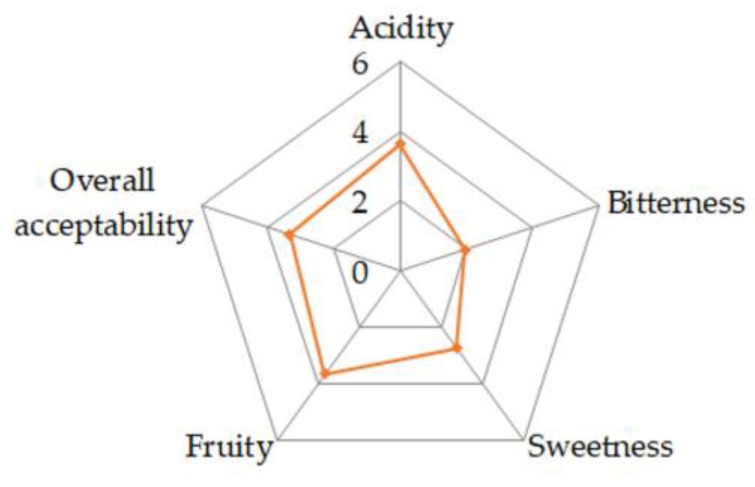
Gustatory profile of kiwi-based drink for the trial in batch fermented with *Torulaspora delbrueckii* UMY196.Data were obtained from medians of the scores indicated by the judges.

The fermentation was interrupted with a residual sugar content of 31.1 ± 8.8 g/L, in agreement with the sugar amount found in the flask tests (Table 1), and with an ethanol value of 7.6 ± 0.1% (v/v) (Table 2). The tasting showed that batch and flask drinks were comparable (Figure 3).

#### 2.2.2. Aroma Profile

The contents of the aromatic compounds were monitored in the must/kiwi juice mix and during the AF, on the third, seventh, and 17th days (decanting), allowing us to follow the evolution of aromas. Fifty-seven free aroma compounds were detected (Table 3). Except for aldehydes, the fermentation was fundamental for the aromatic complexity of the drink [16]. In fact, most of the free aromas were already found in the must/kiwi juice mix, and 33 free aromas (out of 57 compounds detected) increased just on the third day. Their concentrations were relatively unchanged during the fermentation with the exception of 3,4-dimethyl pentanol, phenylethyl acetate, ethyl hexadecanoate, ethyl hydrogen succinate, and *cis*-linalool oxide, for which higher amounts were found on the seventh day. For some of the free aromas, the slight decreases observed may be caused by the bottle opening for the sampling and, except for aldehydes and acids, they were not statistically significant. The most powerful odorants of the kiwi-based drink were ethyl octanoate, phenylethanal, ethyl hexanoate, vinyl-guaiacol, benzaldehyde, and nonanal, for which the odor activity values (OAVs) were 21.1, 3.3, 2.6, 2.2, 1.9, and 1.6, respectively. Ethyl octanoate and ethyl hexanoate are esters derived from the enzymatic activity of yeasts, confirming that the use *of T. delbrueckii* can improve the aromatic profile as occurs in wines [10,16,17]. Comparing the aromatic composition of wines obtained from Cabernet Sauvignon grapes and one of two kiwi fruit juices and purees [18,19], we can hypothesize that phenylethanal and nonanal derive from kiwi juice, as they were not present in Cabernet Sauvignon wines analyzed by the cited authors. All the other compounds originated from the fermentative activity of yeasts, since varietal aromas specific to the Cabernet Sauvignon variety were not detected. This highlights that kiwi juice can positively influence the aromatic profile of the final grape/kiwi-based drink.

Twenty glycosylated aroma compounds were detected and are listed in Table 4. Their concentrations decreased from the beginning of AF, in particular those of benzyl alcohol, 2-hexanal, 4-vinyl guaicol, and 3-oxo-α-ionol. Our findings suggest that the strain of *T. delbrueckii* used for the AF could perform a glycosidase activity, as reported in the literature [27]. Further investigation will be carried out to confirm this activity.

### 2.3. Microvinification

#### 2.3.1. Fermentation Trend and Chemical Composition

The Cabernet Sauvignon/kiwi blend used for the microvinification experiments was characterized by slightly lower contents of sugar and tartaric acid (Table 5) in comparison to the mixture used for the batches and flasks trials; on the other hand, pH and total acidity resulted comparable. Possibly due to the fermentation temperature set at 18 ± 1 °C, the trend of fermentation showed a more prolonged lag phase; indeed, only after five days of fermentation, a similar amount of CO_2_/L to that reached after 48 h in batch experiments was released (27.5 g CO_2_/L). However, once the AF started, the trend was comparable with fermentations in flask and in batches (data not shown).

#### 2.3.2. Chemical Composition of Kiwi-Based Drink

The final product showed a residual sugar content equal to 8.6 ± 0.7 g/L, lower than the predetermined target of 30 g/L (Table 5 *vs* Table 2) and, consequently, with a higher ethanol concentration (9.5 ± 0.2% (*v*/*v*)) in comparison to the flask and batch tests. Contrarily to what was found in the previous trials, a significant increase in total acidity was not detected; it was maintained at a value comparable to the grape/kiwi juice mixes (Table 5). Furthermore, contrarily to flask and batch experiments, succinic acid failed to be produced. The concentration of methanol was 75 mg/L, lower than the law limit for wine fixed as 200 mg/L by the European Community [28].

#### 2.3.3. Sensory Analysis

The descriptive–quantitative profile was evaluated for the final product from microvinification. Moreover, in order to reach the target amount of sugars (30 g/L), reducing the perception of acidity, the drink produced with microvinification was supplemented with 22.4 g/L sucrose. Since the aromas were already produced after three days of fermentation, it is plausible that the addition of sugar at this step could attenuate the perception of acidity and affect the aromatic profile to a relatively low extent. Nevertheless, further steps of scaling-up will optimize the technological operation to stop the fermentation at the required sugar content. The two kiwi-based drinks were tasted by an expert panel that identified the following descriptors: coppery color for the visual perception, peach, floral, passion fruit, and honey for the olfactory perception, and citrus, fruity, apple, and fruit salad for the retro-olfactory perception. Sweetness, acidity, bitterness, olfactory flavor intensity, viscosity, and aromatic intensity were also evaluated.

**Figure 4:**
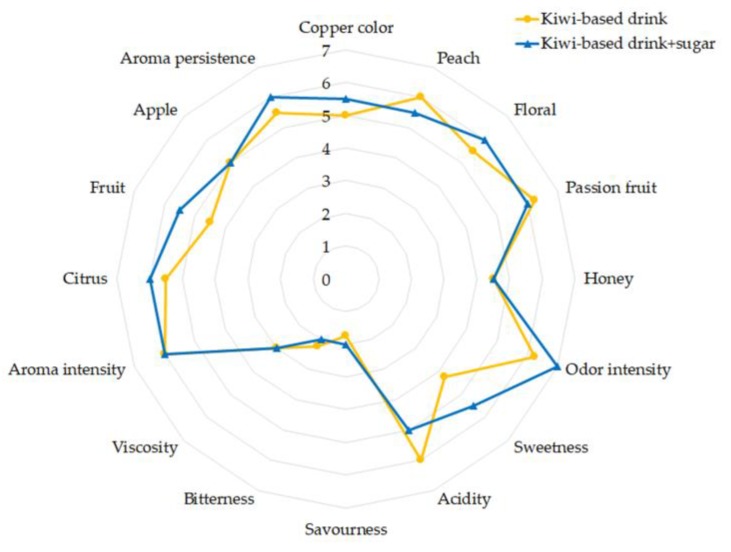
Descriptive–quantitative profile of kiwi-based drink for the trial in a glass demijohn fermented with *Torulaspora delbrueckii* UMY196.

The selected descriptors resulted in accordance with the volatile compounds detected. Indeed, the fruity, honey and floral, apple and peach, and citrus notes, associated with ethyl octanoate, phenylethanal, ethyl hexanoate, and nonanal compounds, respectively, showed the higher OAVs (Section 2.2.2). Figure 4 highlights that the kiwi-based drinks had high olfactory and flavor intensities, indicating their aromatic richness and complexity. The kiwi-based drink added with sugar showed higher perceptions of sweetness, olfactory intensity, citrus and fruity notes, and minor acidity. However, these differences were not statistically significant, indicating the aromatic characteristics of the kiwi-based drink were not affected by its sweetness, as well as its complexity and richness, which were independent of the sugar content.

#### 2.3.4. Consumer Acceptability

**Figure 5:**
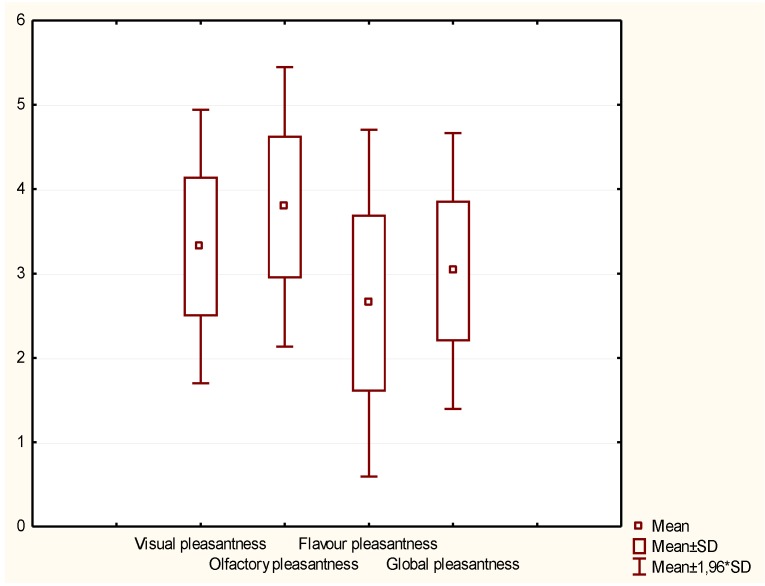
Box and wisker plot of the consumer acceptability test obtained for the kiwi-based drink for the trial in a glass demijohn fermented with *Torulaspora delbrueckii* UMY196.

The acceptability test was performed considering 100 consumers, asking them to judge both pleasantness and acceptability. The panel mainly constituted judges who are used to buying alcoholic beverages (72%) and drinking them at least once a week (65%). As Figure 5 showed, the Cabernet Sauvignon/kiwi drink was appreciated from the olfactory point of view (score 4/5). However, its flavor resulted unpleasant (score 2/5) due to the acidity and the lack of sweetness. Nevertheless, the score of global pleasantness was 3/5 as an average. In general, 67% of consumers considered the product acceptable and 39% stated they would buy it.

## 3. Materials and Methods

### 3.1. Preparation of Fruit Juices and Musts

The two musts used in this study were industrially produced from Chardonnay and Cabernet Sauvignon grapes in two different wineries (Lombardia region, north of Italy). Chardonnay must was employed for the preliminary experiment; it was obtained in vintage 2015 using a Velvet 80 pneumatic press (DIEMME Enologia, Lugo, RA, Italy) working under nitrogen flow and without SO_2_, left for 12 h at 4 °C for settling and then stored at −18 ± 1 °C. The Cabernet Sauvignon must was produced in vintage 2015 for the preliminary and batch experiments and in vintage 2017 for the microvinification. The grape was crushed without sulfur dioxide (SO_2_) and the grape skins were left in the must for three days. The liquid musts were collected and stored at −18 ± 1 °C.

The fruit juices were obtained from cherry (*Prunus avium* var. Durone nero di Vignola), kiwi (*Actinidia chinenesis* var. Gold), peach (*Prunus persica* var. Nucipersica), and strawberry (*Fragaria ananassa* var. Nabila). In the case of kiwi, a further amount of juice was produced for microvinification experiments. The ripe fruits of Italian origin were collected at a local farmer market and they were washed, gently dried, and cut (prior to seed removal) or peeled after purchasing. The fruit juices were obtained with a juicer and stored at −18 ± 1 °C.

### 3.2. Yeast Strains

Pure cultures of *Saccharomyces cerevisiae* Lalvin EC-1118 (EC1118) or *Torulaspora delbrueckii* UMY196 were used for the fermentation trials. *S. cerevisiae* EC1118 is a commercial yeast strain commonly employed for vinification (Lallemand Inc., Montreal, Quebec, Canada). *T. delbrueckii* UMY196 is part of the yeast culture collection of the University of Milan (Italy), and it was isolated from wine. Cells were maintained in yeast extract peptone dextrose (YPD) medium (10 g/L yeast extract, 20 g/L peptone, 20 g/L glucose, pH 5.5) supplemented with 20% (*v*/*v*) glycerol at −80 °C. Cell pre-cultures were obtained by inoculating 1% (*v*/*v*) glycerol stock freeze culture in YPD broth maintained at 30 °C for 24–48 h in aerobiosis. The final biomass was determined by optical density (OD) at 600 nm. For the inocula, cells from the pre-culture were centrifuged at 3500 rpm for 15 min (Hettich, ROTINA 380R, Tuttlingen, Germany), collected, and washed once with 0.9% (*w*/*v*) NaCl. The grape/fruit musts were inoculated at 0.1 ± 0.05 OD 600 nm corresponding to about 1 × 10^6^ CFU/mL.

### 3.3. Fermentation Trials

Grape/fruit-based drinks were produced through alcoholic fermentation (AF). Must/fruit juice mixes were tested in proportions of 80:20 (*v*/*v*) and 60:40 (*v*/*v*) for each must and juice collected, for a total of 48 grape/fruit-based drinks. The level of readily assimilable nitrogen (RAN) was measured in must/fruit juice mixes. If necessary, ammonium sulfate was added prior to the yeast inoculum adjusting the RAN content at 200 mg/L. The AF was carried out in a flask (200 mL) at 25 ± 1 °C and it was daily monitored by weight loss until no weight change was observed after two consecutive days. At the end of AF, the drinks were centrifuged at 5000× *g* for 20 min at 10 °C (Beckman, CA, USA), removing the yeast cells, followed by the addition of potassium metabisulfite (50 mg/L), before being stored at 4 ± 1 °C.

Based on the sensory evaluation, triplicate fermentation of the most pleasant grape/fruit-based drink was carried out in batches (600 mL) at 25 ± 1 °C and in a glass demijohn (4 L) at 18 ± 1 °C, following the same procedure described for the flask trials.

### 3.4. Microbial and Chemical Analysis

The cell enumeration, content of sugars (glucose and fructose), total acidity, pH, and organic acids were determined in both must/fruit juice mix and grape/fruit-based drinks, while ethanol was also evaluated in grape/fruit-based drinks. The ethanol yield (%) was calculated as the molar ratio between the consumed sugars and ethanol produced.

Colony-forming unit (CFU/mL) enumeration was obtained for yeasts and lactic acid bacteria (LABs). The spreading dual plating (100 µL) of useful decimal serial dilutions was carried out on Wallerstein Laboratory (WL) nutrient agar medium (Scharlau, Spain), after 2–4 days of incubation time at 30°C in aerobiosis for yeasts. LABs were enumerated on De Man, Rogosa and Sharpe (MRS; (BD Difco, Thermo-Fisher Scientific, Waltham, MA, USA) solid medium (1.5% *w*/*v* agar agar) at 6.2 pH, supplemented with 20% (*v*/*v*) apple juice and 0.01% (*w*/*v*) cycloheximide; cells were grown in anaerobic conditions at 30 °C for 7–10 days.

Ethanol, glucose, and fructose were determined using a Megazyme ethanol, d-fructose/d-glucose assay kit according to the manufacturer’s instructions. The total acidity was determined by titration up to pH 7 in accordance with the method OIV-MA-AS313-01 [29]. The RAN was quantified by the formol number with titration at pH 8.5 [30]. The quantification of methanol was carried out by Enoconsulting (Erbusco, BS, Italy), an ISO 9000-accredited laboratory, through gas chromatography coupled with a flame ionization detector. The organic acids were quantified as described by Falqué López and Fernández Gómez [31] with some modifications. An Acquity HClass UPLC (Waters, Milford, MA, USA) system equipped with a photo diode array detector 2996 (Waters) was used. Chromatographic separations were performed with a Hypersil BDS C8 250 × 4.6 mm, 5 μm particle size (Alltech, Deerfield, IL, USA). The separation was carried out in isocratic conditions using sulfuric acid (0.01 N) at a flow rate of 0.8 mL/min, and the column temperature was 25 °C. Calibration curves were obtained for tartaric, malic, lactic, citric, acetic, and succinic acids at concentrations of 0.1–10 g/L. Quantification was performed according to the external standard method. Data acquisition and processing were carried out with Empower 2 software (Waters) at 210 nm. The free and glycoconjugate aromas were determined as reported by Fracassetti, Gabrielli, Corona, and Tirelli [32]. Based on the known perception thresholds, the odor activity values (OAVs) were calculated as the ratio between the aroma concentration and its perception threshold.

### 3.5. Sensory Analysis and Acceptability of Grape/Fruit-Based Drinks

The sensory analysis was carried out for each grape/fruit-based drink obtained in flask trials and batch experiments. For the latter, the aroma profile was evaluated during the AF for a total of four samplings. Qualitative sensory analysis and the test of consumer acceptance were carried out for the experiment in a glass demijohn.

For flask and batch experiments, a panel of eight experienced judges (five females, three males) was enrolled. The scored descriptors included acidity, sweetness, bitterness, fruitiness, and the overall acceptability using a 10-cm line scale.

For microvinification in a demijohn, a panel composed of 10 experienced judges (five females, five males) identified the attributes by the consensus method [33], which were scored by a nine-point scale with nine being the highest intensity. The quantitative profile was performed for the kiwi-based drink with and without added sugar. The discriminant capacity of the judges was set at 20% and the replicability was set at 75%.

The test of consumer acceptability was carried out on the kiwi-based drink considering 100 consumers. A five-point scale was used with five being the highest intensity. The attributes were related to the visual, olfactory, taste, and global pleasantness, including the overall acceptability. Further questions were related to the eventual purchase of the beverage tasted and the indication of additional comments.

### 3.6. Statistical Analysis

One-way ANOVA was determined using SPSS Win 12.0 program (SPSS Inc., Chicago, IL). The equations of the calibration curves were assessed by linear regression analysis. Differences were evaluated by the *t*-test, and the significances were set at a value of *p* < 0.05.

## 4. Conclusions

The present study proposes an innovative alcoholic beverage obtained by co-fermenting, using *T. delbrueckii* yeast, Cabernet Sauvignon must and kiwi juice in a proportion of 60:40. To the best of our knowledge, this is the first time such a product appears in literature. The feasibility of co-fermenting grape must and fruit juice was proven, leading to the production of a novel food resulting acceptable from the sensory point of view. The acceptability and approval expressed by potential consumers suggests that this drink could represent a valid strategy for the “re-use” of both kiwi and grape/must surplus, combining a sustainable approach with innovation in the alcoholic beverage field. The production of this alcoholic beverage can provide benefits to both wineries and farmers, since the losses of grape/wine and fruit can be limited and a longer use of the fermentation compartment of the cellar can be achieved. The proposed Cabernet Sauvignon/kiwi drink can be considered a pleasant beverage with low alcohol content, indicated as an aperitif. Future developments foresee a scale-up in the production of this drink, eventually using the equipment already present in a cellar to produce kiwi juice without compromising the quality of the juice, and managing the fermentation in order to preserve the desired residual sugar attenuating the acidity.

## Figures and Tables

**Table 1:** Chemical parameters determined for fermentation trial with grape must/kiwi juice fermented with *Saccharomyces cerevisiae* EC1118 (S. c.) and *Torulaspora delbrueckii* UMY196 (T. d.).

Must	Proportion *		Sugar (g/L)		Ethanol (*v*/*v*)		Total Acidity (g Tartaric Acid/L)	
Fermenting Yeast			*S. c.*	*T. d.*	*S. c.*	*T. d.*	*S. c.*	*T. d.*
Cabernet Sauvignon	80:20	T0	180 ± 17	196 ± 18	-	-	7.8 ± 0.3	7.5 ± 0.3
		EF	0.80 ± 0.0	71 ± 2	10.8 ± 0.5	6.5 ± 0.3	8.6 ± 0.7	11.5 ± 0.7
	60:40	T0	153 ± 14	169 ± 16	-	-	9.5 ± 0.4	10.1 ± 0.4
		EF	0.10 ± 0.0	52 ± 17	9.6 ± 0.3	7.0 ± 1.3	11.0 ± 0.2	15.0 ± 0.5
Chardonnay	80:20	T0	147 ± 14	160 ± 15	-	-	9.3 ± 0.4	9.2 ± 0.4
		EF	0.15 ±0.07	0.33 ± 0.00	8.7 ± 0.2	9.1 ± 0.1	10.2 ± 0.4	13.9 ± 0.5
	60:40	T0	127 ± 12	135 ± 13	-	-	11.1 ± 0.4	11.4 ± 0.5
		EF	0.05 ± 0.02	3.2 ± 2.6	7.6 ± 0.1	7.4 ± 0.3	12.2 ± 0.2	16.3 ± 0.3

* The proportion is related to must/kiwi juice (*v*/*v*). T0: concentrations of chemical parameters in kiwi juice/must; EF: concentrations of chemical parameters in grape/kiwi drink at the end of alcoholic fermentation.

**Table 2:** Chemical parameters determined for batch fermentation with Cabernet Sauvignon must/kiwi juice 40:60 (*v*/*v*) fermented with *Torulaspora delbrueckii* UMY196.

Chemical Parameter	Must/Kiwi Juice	Kiwi-Based Drink
Sugar (g/L)	190.1 ± 8.0	31.1 ± 8.8
Ethanol (*v*/*v*)	-	7.6 ± 0.4
pH	3.2 ± 0.0	3.2 ± 0.1
Total acidity (g tartaric acid/L)	10.2 ± 0.3	14.9 ± 1.4
Tartaric acid (g/L)	1.70 ± 0.06	1.66 ± 0.05
Malic acid (g/L)	2.58 ± 0.39	3.00 ± 0.22
Lactic acid (g/L)	n.d.	n.d.
Acetic acid (g/L)	n.d.	0.21 ± 0.06
Citric acid (g/L)	6.03 ± 0.22	5.86 ± 0.14
Succinic acid (g/L)	n.d.	1.83 ± 0.11

The trial was carried out in triplicate (volume: 0.6 L); n.d.: not detected.

**Table 3:** Evolution of free aromatic compounds determined for the batch fermentation with Cabernet Sauvignon must/kiwi juice 60:40 (*v*/*v*) fermented with *Torulaspora delbrueckii* UMY196.

Compound	Perception Threshold (μg/L)	Descriptor	Cabernet Sauvignon Must/Kiwi Juice	Days of Fermentation		
				3	11	17 *
*Acids*						
Isobutyric acid	2300 ^c^	Rancid, butter, cheese	4.12 ± 1.04	89.77 ± 78.06	n.d.	n.d.
Isopentanoic acid	33 ^e^	Sweat, rancid	n.d.	100.72 ± 95.63	35.92 ± 2.95	36.41 ± 6.74
Pentanoic acid	17 ^d^	Sweat	n.d.	3.84 ± 0.94	3.00 ± 1.25	2.00 ± 0.59
Hexanoic acid	420 ^c^	Sweat	32.02 ± 11.50	603.73 ± 57.96	465.00 ± 51.79	388.51 ± 33.64
*trans*-2-Hexenoic acid	-	Must, fat	25.31 ± 8.52	42.69 ± 10.35	35.55 ± 5.43	27.84 ± 2.18
Octanoic acid	500 ^c^	Cheese, sweat	13.13 ± 0.87	563.38 ± 15.85	479.39 ± 43.96	455.36 ± 29.24
Decanoic acid	1000 ^d^	Rancid, fat	n.d.	319.58 ± 32.81	155.78 ± 41.90	112.86 ± 54.34
9-Decenoic acid	2 ^d^	Fat	n.d.	112.98 ± 64.23	79.20 ± 14.41	71.54 ± 33.18
2-Methylbutanoic acid	33 ^d^	Cheese, sweat	n.d.	130.16 ± 21.04	125.10 ± 16.46	89.07 ± 21.52
2-Butenoic acid	-	Milky	52.51 ± 9.48	72.45 ± 8.05	76.33 ± 22.06	60.12 ± 8.34
**Total**			**127.09 ± 31.41**	**2039.31 ± 384.93**	**1455.26 ± 200.20**	**1243.71 ± 189.78**
*Alcohols*						
Isobutanol	40,000 ^d^	Wine, solvent, bitter	60.27 ± 9.53	138.66 ± 17.84	94.70 ± 18.77	76.05 ± 7.76
3-Penten-2-ol	-	Green, vinyl	7.70 ± 1.42	32.98 ± 15.11	32.91 ± 7.54	27.04 ± 6.73
1-Pentanol	-	Balsamic	n.d.	10.62 ± 1.70	6.72 ± 1.80	5.83 ± 0.96
1-Hexanol	1110 ^g^	Resin, flower, green	48.55 ± 15.96	396.08 ± 70.77	328.74 ± 23.61	351.68 ± 56.33
2-Hexanol	-	Resin, flower, green	20.77 ± 5.06	29.54 ± 3.40	26.19 ± 4.83	24.54 ± 0.64
3-Ethoxy-1-propanol	-	Fruit	n.d.	184.45 ± 28.60	169.08 ± 4.77	152.96 ± 19.25
*cis*-3-Hexen-1-ol	400 ^e^	Grass	29.70 ± 10.07	35.87 ± 2.94	25.83 ± 0.50	20.34 ± 2.47
2-Ethyl-1-decanol	-	Fat	25.65 ± 4.03	20.32 ± 14.26	2.21 ± 0.48	2.29 ± 0.21
4-Hepten-1-ol	-	Green, grassy odor	n.d.	4.66 ± 4.47	4.54 ± 2.23	5.25 ± 0.92
Isoamyl alcohol	30,000 ^d^	Spirit, alcoholic	52.30 ± 6.27	16569 ± 1348	12909 ± 860	11224 ± 953
2-Methyl-4-octanol	-	Cucumber	n.d.	4.85 ± 1.19	2.72 ± 0.89	4.28 ± 0.44
2,3-Butanediol	-	Fruit, onion	n.d.	2.49 ± 1.62	11.07 ± 2.17	10.71 ± 0.78
Linalool	15 ^d^	Flower, lavender	n.d.	8.39 ± 4.80	2.59 ± 0.55	2.78 ± 0.40
3,4-Dimethyl pentanol	-	-	2.95 ± 1.24	2.23 ± 0.25	107.98 ± 11.40	92.08 ± 15.65
α-Terpineol	250 ^d^	Oil, anise, mint	8.08 ± 1.04	10.54 ± 2.10	9.31 ± 2.06	9.93 ± 1.50
2-Phenyl-2-hexanol	-	-	n.d.	6.33 ± 3.12	3.78 ± 0.83	4.65 ± 0.82
Citronellol	100 ^d^	Rose	n.d.	3.26 ± 0.46	3.37 ± 0.84	3.72 ± 1.37
Geraniol	30 ^d^	Rose, geranium	n.d.	9.32 ± 2.71	8.61 ± 0.97	8.54 ± 1.08
2-Phenylethanol	10,000 ^d^	Honey, spice, rose, lilac	n.d.	15978 ± 504	16286 ± 1805	14660 ± 690
*p*-Tyrosol	-	-	n.d.	258.13 ± 59.94	271.51 ± 20.35	223.25 ± 13.96
**Total**			**255.97 ± 54.62**	**33704 ± 2089**	**30307 ± 2770**	**26256 ± 2174**
*Aldehydes*						
Nonanal	8 ^d^	Fat, citrus, green	66.71 ± 22.51	12.21 ± 2.44	10.99 ± 1.30	12.45 ± 3.18
Benzaldehyde	5 ^f^	Almond, sugar	16.27 ± 3.80	7.78 ± 3.27	7.10 ± 1.14	9.26 ± 4.13
Phenylethanal	1 ^e^	Honey, sweet, hawthorn	n.d.	5.73 ± 2.88	4.27 ± 0.40	3.25 ± 1.24
2,4-Dimethyl benzaldehyde	-	Sweet	4.37 ± 1.11	4.25 ± 0.53	6.41 ± 2.61	7.23 ± 2.46
**Total**			**87.35 ± 27.42**	**29.97 ± 9.12**	**28.77 ± 5.45**	**32.19 ± 11.00**
*Benzenoids*						
4-Vinyl guaiacol	40 ^d^	Clove, curry	8.84 ± 1.91	100.32 ± 3.32	108.49 ± 12.39	83.19 ± 8.35
Guaiacol	9.5 ^e^	Smoke, sweet, medicine	4.20 ± 2.21	10.45 ± 2.97	9.22 ± 1.42	6.10 ± 3.94
Syringol	-	Medicine, phenol, smoke	88.84 ± 24.29	65.25 ± 2.79	61.30 ± 12.28	51.05 ± 7.30
**Total**			**101.88 ± 28.41**	**176.01 ± 9.08**	**179.01 ± 26.09**	**146.28 ± 19.59**
*Esters*						
Isoamyl acetate	12,270 ^d^	Banana	n.d.	19.52 ± 2.66	17.18 ± 1.29	21.81 ± 3.48
Ethyl hexanoate	14 ^a^	Apple, peach	n.d.	59.77 ± 3.26	37.93 ± 6.20	35.84 ± 3.75
Ethyl octanoate	2 ^f^	Fruit, fat	n.d.	47.79 ± 7.05	44.75 ± 1.55	42.12 ± 2.66
Ethyl decanoate	200 ^d^	Grape	5.15 ± 1.04	105.10 ± 57.31	36.54 ± 10.77	29.11 ± 16.62
Diethyl succinate	200,000 ^e^	Wine, fruit	n.d.	6.21 ± 2.14	18.72 ± 2.34	26.21 ± 2.96
Ethyl-9-decenoate	-	Fruity	n.d.	45.76 ± 27.65	26.45 ± 14.37	29.94 ± 12.11
Ethyl acetate	7500 ^d^	Pineapple	n.d.	28.19 ± 11.67	15.78 ± 4.43	14.21 ± 2.03
α-Isoamyl-γ-butyrolactone	-	Coumarin, sweet	n.d.	33.19 ± 6.15	35.56 ± 6.72	30.75 ± 3.19
Phenylethyl acetate	250 ^d^	Rose, honey, tobacco	n.d.	77.32 ± 10.94	109.03 ± 18.45	118.19 ± 18.44
Butyl isobutyrate		Fruity, green, apple, banana	n.d.	112.44 ± 13.31	100.93 ± 15.08	82.38 ± 9.51
γ-Nonalactone	25 ^d^	Coconut, peach	4.71 ± 2.87	5.29 ± 0.87	6.24 ± 0.99	6.92 ± 0.26
Diethyl malate	-	Brown sugar, sweet	n.d.	7.05 ± 2.25	7.47 ± 0.63	8.57 ± 1.56
Methyl hexadecanoate	-	Fat, wax	44.59 ± 6.38	40.93 ± 2.45	37.31 ± 4.58	33.74 ± 5.44
Ethyl hexadecanoate	-	Wax	n.d.	24.95 ± 10.29	70.72 ± 16.86	53.35 ± 19.71
Ethyl hydrogen succinate	-	Wine, fruit	n.d.	43.36 ± 29.15	99.77 ± 15.86	87.70 ± 19.43
Phenethyl propionate	-	Fruit	n.d.	18.16 ± 2.35	15.54 ± 2.32	14.09 ± 1.21
**Total**			**54.46 ± 10.28**	**675.03 ± 189.51**	**679.92 ± 122.45**	**634.95 ± 122.37**
*Furanoids*						
*cis*-Linalool oxide	-	Flower	n.d.	1.96 ± 0.50	197.44 ± 27.48	175.09 ± 4.73
*Ketones*						
6-Methyl-2-heptanone	-	Soap	n.d.	8.16 ± 4.18	6.73 ± 4.97	6.01 ± 1.16
*Norisoprenoids*						
3-Hydroxy-β-damascone	-	Apple, tea, tobacco	11.31 ± 6.25	146.28 ± 106.57	65.23 ± 30.63	87.69 ± 8.09
*Thiols*						
3-(Methylthio)-propanol	1000 ^b^	Sweet, potato	n.d.	51.62 ± 8.23	63.92 ± 6.73	53.89 ± 6.25

The trial was carried out in triplicate. * Sampling at the end of fermentation; n.d.: not detected. References: ^a^ [20]; ^b^ [21]; ^c^ [22]; ^d^ [23]; ^e^ [24]; ^f^ [25]; ^g^ [26].

**Table 4:** Evolution of glycosylated aromatic compounds determined for the batch fermentation with Cabernet Sauvignon must/kiwi juice 60:40 (*v*/*v*) fermented with *Torulaspora delbrueckii* UMY196.

Compound	Perception Threshold (μg/L)	Descriptor	Cabernet Sauvignon Must/Kiwi Juice	Days of Fermentation		
				3	11	17 *
*Acids*						
Nonanoic acid	-	Green, fat	20.20 ± 1.22	11.13 ± 1.23	17.10 ± 2.61	17.89 ± 3.22
Geranic acid	-	Green, floral	28.77 ± 9.87	26.22 ± 10.76	21.65 ± 10.08	20.65 ± 2.81
**Total**			**48.97 ± 11.09**	**37.25 ± 11.99**	**38.75 ± 12.69**	**38.54 ± 6.03**
*Alcohols*						
3-Penten-2-ol	-	Green, vinyl	41.32 ± 2.79	41.41 ± 4.60	30.93 ± 9.42	38.67 ± 6.20
1-Hexanol	1110^c^	Resin, flower, green	34.95 ± 4.95	33.81 ± 1.92	32.93 ± 5.49	28.73 ± 1.36
2-Hexanol	-	Resin, flower, green	27.31 ± 2.96	24.95 ± 0.83	25.61 ± 5.46	26.56 ± 3.20
3-Octanol	-	Moss, nut, mushroom	79.09 ± 4.60	98.55 ± 4.14	99.64 ± 2.55	106.25 ± 2.74
Linalool	15^a^	Flower, lavender	12.10 ± 2.69	11.09 ± 1.49	10.19 ± 0.78	7.87 ± 3.44
α-Terpineol	250^a^	Oil, anise, mint	9.34 ± 1.07	7.99 ± 0.71	9.21 ± 0.40	10.16 ± 1.85
Nerol	-	Sweet	112.53 ± 5.18	125.19 ± 8.13	130.25 ± 4.49	131.72 ± 13.58
Benzyl alcohol	-	Sweet, flower	334.71 ± 13.38	113.71 ± 6.47	110.66 ± 8.20	78.00 ± 9.63
8-Hydroxygeraniol	-	-	8.34 ± 1.82	8.38 ± 1.22	8.55 ± 0.63	16.24 ± 3.92
**Total**			**659.69 ± 39.46**	**465.08 ± 29.51**	**457.97 ± 37.41**	**444.20 ± 45.92**
*Aldehydes*						
2-Hexanal	-	Grass, tallow, fat	164.99 ± 19.71	42.11 ± 7.92	34.88 ± 8.33	33.52 ± 5.48
Nonanal	8^a^	Fat, citrus, green	4.31 ± 1.16	3.08 ± 0.19	2.45 ± 0.57	12.78 ± 2.33
Benzaldehyde	5^b^	Almond, sugar	9.22 ± 2.66	4.09 ± 2.22	2.63 ± 0.61	2.52 ± 0.91
**Total**			**178.51 ± 23.54**	**49.28 ± 10.34**	**39.96 ± 9.52**	**48.82 ± 8.72**
*Benzenoids*						
4-Vinyl guaiacol	40^a^	Clove, curry	154.10 ± 36.96	84.51 ± 13.28	100.87 ± 10.01	95.61 ± 5.82
Eugenol	-	Clove, honey	14.20 ± 2.32	17.10 ± 2.98	16.96 ± 1.85	17.52 ± 0.27
Syringol	-	Medicine, phenol, smoke	49.85 ± 19.09	31.19 ± 8.11	58.55 ± 22.44	44.61 ± 9.42
**Total**			**218.16 ± 58.37**	**132.80 ± 24.36**	**176.38 ± 34.29**	**157.73 ± 15.52**
*Norisoprenoids*						
3-oxo-α-damascone	-	Apple	35.24 ± 11.16	39.86 ± 1.83	38.49 ± 3.05	37.47 ± 3.72
3-Oxo-α-ionol	-	Spice, tea, tobacco	118.34 ± 32.57	83.13 ± 27.57	81.43 ± 2.23	70.78 ± 8.83
**Total**			**153.58 ± 58**	**123.00 ± 29.39**	**119.92 ± 5.28**	**108.25 ± 12.55**
*Furanoids*						
*cis*-Linalool oxide	-	Flower	11.18 ± 1.72	11.34 ± 1.09	12.41 ± 0.24	12.26 ± 0.82

The trial was carried out in triplicate. * Sampling at the end of fermentation; n.d.: not detected. References: ^a^ [23]; ^b^ [25]; ^c^ [26].

**Table 5:** Chemical parameters determined for the microvinification trial with Cabernet Sauvignon must/kiwi juice 60:40 (*v*/*v*) fermented with *Torulaspora delbrueckii* UMY196.

Chemical Parameter	Must/Kiwi Juice	Kiwi-Based Drink
Sugar (g/L)	180 ± 17	8.6 ± 0.7
Ethanol (*v*/*v*)	-	9.5 ± 0.2
Methanol (mg/L)	-	75.0
pH	3.3 ± 0.01	3.5 ± 0.01
Total acidity (g tartaric acid/L)	9.2 ± 0.4	9.4 ± 0.6
Tartaric acid (g/L)	0.79 ± 0.03	0.51 ± 0.10
Malic acid (g/L)	4.09 ± 0.29	2.63 ± 0.54
Lactic acid (g/L)	n.d.	n.d.
Acetic acid (g/L)	n.d.	0.11 ± 0.07
Citric acid (g/L)	5.37 ± 0.28	6.05 ± 0.81
Succinic acid (g/L)	n.d.	n.d.

The trial was carried out in triplicate; n.d.: not detected.

## Supplementary Materials

The following are available online at https://www.mdpi.com/2218-1989/9/5/86/s1: Table S1: Ethanol yield in the flask fermentation trials fermented with *Saccharomyces cerevisiae* EC1118 (S. c.) and *T. delbrueckii* UMY196 (T. d.); Table S2: Chemical parameters determined for the fermentation trial with must/cherry juice fermented with *S. cerevisiae* EC1118 (S. c.) and *T. delbrueckii* UMY196 (T. d.); Table S3: Chemical parameters determined for the fermentation trial with must/peach juice fermented with *S. cerevisiae* EC1118 (S. c.) and *T. delbrueckii* UMY196 (T. d.); Table S4: Chemical parameters determined for the fermentation trial with must/strawberry juice fermented with *Saccharomyces cerevisiae* EC1118 (S. c.) and *T. delbrueckii* UMY196 (T. d.).

## Appendix A

**Table A1:** Summary of the chemical parameters (residual sugars, ethanol, pH, total acidity, organic acid profile) for the must/fruit-based drink. The data related to organic acids are reported in Figure A1.

Fruit-Based Drink	Chemical Characteristic of the Final Product
Cherry-based drinks (Table S2)	Residual sugars: <1.0 ± 0.0 g/L in all the combinations. Ethanol: It was the highest compared to the others investigated combinations, reaching the maximum in combination with Cabernet Sauvignon 80:20 (12.7 ± 0.1% (*v*/*v*) for *S. cerevisiae* and 12.3 ± 0.7% (*v*/*v*) for *T. delbrueckii*). pH: 3.27 ± 0.02 to 3.54 ± 0.00. Total acidity: Higher in beverages produced with *T. delbrueckii*. Organic acids: Malic acid decreased during the alcoholic fermentation, although it was the major organic acid in the drinks produced. Acetic acid was found only in the drinks fermented with *T. delbrueckii *(maximum 1.8 ± 0.0 g/L in the 80:20 mix with Cabernet Sauvignon).
Kiwi-based drinks (Table 1)	Residual sugars: Higher in the mixes Cabernet Sauvignon fermented by *T. delbrueckii* (71 ± 2 g/L and 52 ± 17 g/L for 80:20 and 60:40 combinations, respectively). Ethanol: 6.5 ± 0.3% (*v*/*v*) and 7.0 ± 1.3% (*v*/*v*) for mixes 80:20 and 60:40, respectively, fermented by* T. delbrueckii *in comparison to the respective fermented drinks with *S. cerevisiae* (10.8 ± 0.5% (*v*/*v*) for 80:20 and 9.6 ± 0.3% (*v*/*v*) for 60:40. pH: Slightly higher in drinks fermented with *T. delbrueckii *(3.13 ± 0.08 to 3.50 ± 0.01) in the mix 60:40 with Chardonnay must. Total acidity: 11.5 ± 0.7 to 16.3 ± 0.3 g tartaric acid/L in the tests with *T. delbrueckii*. Organic acids: The most abundant was citric acid, deriving from kiwi juice. Succinic acid was detected at the end of fermentation, while acetic acid was found only in drinks based on Chardonnay must and fermented by *T. delbrueckii*.
Peach-based drinks (Table S3)	Residual sugars: <1.0 ± 0.0 g/L in all the combinations. Ethanol: Higher in the 80:20 combinations for both musts and yeasts used. pH: Slightly higher in drinks fermented with *T. delbrueckii *(3.30 ± 0.01 to 3.72 ± 0.01) in the mix 60:40 with Chardonnay must. Total acidity: Slight differences between drinks fermented with *S. cerevisiae* and *T. delbrueckii*. Organic acids: Both tartaric and malic acids decreased during the fermentation. The drinks contained succinic acid (highest amount 1.40 ± 0.23 g/L), as well as acetic acid (highest amount 0.98 ± 0.03 g/L).
Strawberry-based drinks (Table S4)	Residual sugars: <2.5 ± 1.5 g/L, except for the trial with Cabernet Sauvignon must at the proportion 80:20 fermented by *T. delbrueckii* in which the residual sugar was 44 ± 5 g/L. Ethanol: From 4.4 ± 1.0% (*v*/*v*) in the trial with Chardonnay must at the proportion 60:40 fermented by *T. delbrueckii* to 9.9 ± 0.9% (*v*/*v*) in the test with Cabernet Sauvignon must at the proportion 80:20 fermented by *S. cerevisiae*. The higher concentrations were detected in drinks obtained from mixes with Cabernet Sauvignon, attributable to the higher sugar content. pH: No significant differences among the different combinations must/strawberry juice (range 2.97 ± 0.00 to 3.22 ± 0.02). Total acidity: Higher in the fermented mixes with *T. delbrueckii*. Organic acids: A significant decrease in tartaric acid occurred. Acetic acid was in the range 1.3 ± 0.0 g/L to 2.4 ± 0.3 g/L, except for the drinks based on Chardonnay must and fermented with T. *delbrueckii*.
