# Supplementary material for: Innovative Alcoholic Drinks Obtained by Co-Fermenting Grape Must and Fruit Juice
Source: Metabolites. 2019 Apr 30;9(5):86. doi: 10.3390/metabo9050086 (PMC6571751; doi:10.3390/metabo9050086)
Supplement: Supplementary file 1 [file metabolites-09-00086-s001.pdf]

Table S1

Ethanol yield in the flask fermentation trials fermented with *S. cerevisiae* EC1118 (*S. c.*) and *T. delbrueckii* UMY196 (*T. d.*). Different letters mean significant difference among different grape must/fruit drink obtained with the same fruit juice. *: significant difference between the two yeast species fermenting the same grape must/fruit juice mix; ns: not significant.

**Must**	**Proportion**		**Cherry**		**Kiwi**		**Peach**		**Strawberry**	
Cabernet Sauvignon	80:20	*S. c.*	91 ± 9^{ab}	ns*	93±3	ns*	98 ± 10^{ab}	ns*	92 ± 3^{a}	ns*
		T. d.	84 ± 4^{ab}		81±2		83 ± 8^{a}		101 ± 13^{a}	
	60:40	*S. c.*	104 ± 7^{b}	ns*	97±2	ns*	109 ± 13^{b}	0.0192*	83 ± 1^{a}	ns*
		T. d.	94 ± 6^{b}		92±3		90 ± 9^{ab}		89 ± 11^{ab}	
Chardonnay	80:20	*S. c.*	86 ± 7^{a}	ns*	92±1	ns*	95 ± 10^{ab}	ns*	112 ± 6^{b}	0.0002*
		T. d.	82 ± 4^{a}		88±1		103 ± 13^{b}		81 ± 12^{b}	
	60:40	*S. c.*	83 ± 8^{a}	ns*	92±1	ns*	92 ± 7^{a}	ns*	122 ± 25^{b}	0.0000*
		T. d.	77 ± 14^{a}		87±1		98 ± 17^{ab}		88 ± 22^{ab}	

Supplementary S2

Chemical parameters determined for the fermentation trial with must/cherry juice fermented with *S. cerevisiae* EC1118 (*S. c.*) and *T. delbrueckii* UMY196 (*T. d.*).

Must	Proportion*		Sugar (g/L)		Ethanol (v/v)		Total acidity (g tartaric acid/L)	
Fermenting yeast			*S. c.*	*T. d.*	*S. c.*	*T. d.*	*S. c.*	*T. d.*
Cabernet Sauvignon	80:20	T0	217±20	227±20	--	--	5.7±0.2	5.4±0.2
		EF	0.44±0.08	0.84±0.23	12.7±0.1	12.3±0.7	6.5±0.1	8.7±0.1
	60:40	T0	177±16	187±17	--	--	6.0±0.2	6.3±0.3
		EF	0.42±0.08	0.12±0.02	11.9±0.4	11.3±0.4	6.7±0.1	9.1±0.7
Chardonnay	80:20	T0	162±15	187±17	--	--	7.5±0.3	6.3±0.3
		EF	0.15±0.03	1.00±0.00	8.9±0.1	9.8±0.5	8.6±0.3	10.8±0.1
	60:40	T0	138±12	173±16	--	--	6.3±0.3	7.2±0.3
		EF	n.d.	0.50±0.23	7.4±0.0	8.5±1.1	6.7±0.1	10.8±0.1

*: the proportion is related to must:cherry juice (v/v).

Legend: T0: concentrations of chemical parameters in kiwi juice/must; EF concentrations of chemical parameters in mixed-cherry wine at the end of alcoholic fermentation; n.d.: not detected.

Supplementary S3

Chemical parameters determined for the fermentation trial with must/peach juice fermented with *S. cerevisiae* EC1118 (*S. c.*) and *T. delbrueckii* UMY196 (*T. d.*).

**Must**	**Proportion***		**Sugar (g/L)**		**Ethanol (v/v)**		**Total acidity (g tartaric acid/L)**	
Fermenting yeast			*S. c.*	*T. d.*	*S. c.*	*T. d.*	*S. c.*	*T. d.*
Cabernet Sauvignon	80:20	T0	187±17	214±19	--	--	4.8±0.2	4.5±0.2
		EF	0.19±0.02	0.10±0.00	11.7±0.3	11.4±0.1	9.3±0.9	7.3±0.3
	60:40	T0	147±13	180±16	--	--	4.4±0.2	4.4±0.2
		EF	0.05±0.03	0.17±0.05	10.3±0.4	10.4±0.1	6.4±0.0	7.5±0.0
Chardonnay	80:20	T0	153±14	153±14	--	--	6.6±0.3	6.9±0.3
		EF	0.10±0.05	0.18±0.03	9.4±0.2	10.2±0.4	8.3±0.3	9.5±0.4
	60:40	T0	140±13	113±10	--	--	6.2±0.2	5.1±0.2
		EF	0.08±0.03	0.08±0.07	8.3±0.3	7.1±0.6	8.3±0.1	8.0±0.4

*: the proportion is related to must:peach juice (v/v).

Legend: T0: concentrations of chemical parameters in kiwi juice/must; EA concentrations of chemical parameters in mixed-peach wine at the end of alcoholic fermentation; n.d.: not detected.

Supplementary S4

Chemical parameters determined for the fermentation trial with must/strawberry juice fermented with *S. cerevisiae* EC1118 (*S. c.*) and *T. delbrueckii* UMY196 (*T. d.*).

Must	Proportion*		Sugar (g/L)		Ethanol (v/v)		Total acidity (g tartaric acid/L)	
Fermenting yeast			*S. c.*	*T. d.*	*S. c.*	*T. d.*	*S. c.*	*T. d.*
Cabernet Sauvignon	80:20	T0	167±15	168±15	--	--	5.9±0.2	5.1±0.2
		EF	n.d.	44±5	9.9±0.9	7.9±0.3	7.5±0	8.8±0.3
	60:40	T0	146±13	151±14	--	--	6.3±0.3	5.3±0.2
		EF	n.d.	2.5±1.5	7.9±1.1	8.5±0.4	6.8±0.3	9.5±0.5
Chardonnay	80:20	T0	92±8	94±8	--	--	5.1±0.2	4.8±0.2
		EF	n.d.	n.d.	6.7±0.3	4.9±0.4	6±0.4	6.9±0
	60:40	T0	64±6	79±7	--	--	5.7±0.2	6±0.2
		EF	n.d.	n.d.	5.0±0.8	4.4±1.0	6.3±0.2	7.7±0.2

*: the proportion is related to must:strawberry juice (v/v).

Legend: T0: concentrations of chemical parameters in kiwi juice/must; EA concentrations of chemical parameters in mixed-strawberry wine at the end of alcoholic fermentation; n.d.: not detected.
